# Society Proceedings

**Published:** 1902-03

**Authors:** 


### SOCIETY PROCEEDINGS.

### PENNSYLVANIA STATE BOARD OF VETERINARY MEDICAL EXAMINERS.

THE semi-annual meeting of the Board was convened December 16 and 17, 1901, in Philadelphia, Pa. Minutes of annual meeting approved as read.

The members present were Drs. Sallade, Ridge, and Hoskins. Absent, Drs. McNeil and Helmer, from whom letters were received explaining their absence.

The Secretary reported that Governor Stone had reappointed W. Horace Hoskins for another term, succeeding himself.

There were three applicants before the Board, namely, Drs. Edward Martin, of Pittsburg, and H. H. Collins, of Elizabethville, who were in attendance on their first examination, and John F. McAnulty, of Philadelphia, a previous applicant before the Board.

Dr. Martin exhibited his diploma of the Royal College of Veterinary Surgeons, 1896; Dr. H. H. Collins, of Toronto, 1892. The papers requisite as to character, etc., were filed.

The examinations were conducted by the several members of the Board present. Licenses were granted to the three applicants.

President Sallade reported the prosecution of Wesley Wilt for violation of the State law, his conviction and punishment by fine and costs and subsequent imprisonment for non-payment of fine.

Cases for prosecution were discussed as existing at Columbia, York, Waynesboro, and Tamaqua, and actions directed to be instituted at once against said violators.

Bill for twenty-five dollars ($25) for expenses in connection with the prosecutions of Wesley Wilt and Jabez Burkes was reported by Dr. Sallade and, on motion, ordered paid. Other bills for expenses of the members were submitted and ordered paid.

W. HORACE HOSKINS,
Secretary.

### THE ILLINOIS VETERINARY AND SURGICAL ASSOCIATION

THIS Association met in annual session at the Brunswick Hotel, Decatur, Ill., on January 9 and 10, 1902. The meeting was called to order by the President of the Association, Dr. V. G. Hunt, of Arcola, Ill.

The opening address of President Hunt was an exceedingly interesting discourse, replete with interest to the Association.

Dr. J. C. Hoxsey, of Auburn, was elected to membership, following a favorable report from the committee upon his application. President Hunt then introduced Dr. Hoxsey to the Society.

The election of officers for the ensuing year was then held and resulted as follows:

For President, Dr. V. G. Hunt, of Arcola, Ill.; First Vice-President, Dr. C. A. Hurlbutt, of Stonington, Ill.; Second Vice-President, Dr. F. Glassbrenner, of Alton, Ill.; Secretary, Dr. W. A. Swain, of Mt. Pulaski, Ill.; Treasurer, Dr. J. M. Reed, of Mattoon, Ill.

Standing committees appointed by the President for the coming year were :

*Committee on Membership.*—Drs. S. H. Swain, J. M. Reed, A. Travis, C. A. Hurlbutt, and W. C. Dawson.

*Committee on Programme.*—Drs. V. G. Hunt, W. A. Swain, and S. H. Swain.

*Committee on Arrangements.*—Drs. V. G. Hunt and F. Glassbrenner.

*Committee on Legislation.*—Drs. John Osborne, V. G. Hunt, S. H. Swain, and C. A. Hurlbutt.

An excellent paper by Dr. J. M. Reed, upon the subject of "Metroperitonitis," brought out some very interesting and instructive points with reference to this disease. He was responded to by Dr. V. G. Hunt.

Owing to the absence of a paper assigned to Dr. R. W. Brathwaite upon "Bovine Tuberculosis," the subject was very generally and well discussed by several of those present. Dr. S. H. Swain reported upon a case of herpes circinatus, being a very extraordinary case, which brought forth some very interesting points in the treatment of this disease.

The second day's session was called to order by President Hunt. Much discussion was indulged in by those present as to what should constitute eligibility of members. On motion any action on the subject was deferred until the next meeting.

An able and instructive paper by Dr. V. G. Hunt, on "Cerebro-spinal Meningitis," was well received, and was then responded to by Drs. John Osborne and S. H. Swain.

Dr. W. A. Swain read a very interesting report of a case of "Summer Sores on the Penis," which brought out considerable discussion as to the various modes of treating this disease.

Among the visitors present was Dr. A. Babb, of Springfield, Ill. On motion the location of meeting was fixed in Decatur, and dates in August to be selected by the Committee on Programme. Meeting adjourned until August, 1902, at Decatur, Ill.

W. A. Swain, V. S.,
Secretary.

### GENESEE VALLEY VETERINARY MEDICAL SOCIETY.

The annual meeting of the Society was held at the Livingston Hotel, Rochester, N. Y., January 17, 1902, it being one of the most interesting in the history of the Society. The meeting was called to order at 11 o'clock by President Dr. T. S. Rich, of Avon, N. Y., and the following members responded to roll-call : W. G. Dodd, Canandaigua ; P. I. Johnson, Williamson ; G. C. Kesler, Holley ; N. N. Loefler, Geneseo ; J. C. McKenzie, Rochester ; T. S. Rich, Avon ; W. E. Stocking, Medina ; J. E. Smith, Webster ; A. George Tegg, Rochester ; Leroy Webber, Rochester ; D. P. Webster, Hilton ; W. J. Payne, Fairport ; A. McConnell, Brockport.

Following the usual routine of business came the election of officers for the ensuing year, which was as follows : President, O. B. French, Honeoye Falls ; Vice-President, G. C. Kesler, Holley ; Secretary, W. E. Stocking, Medina ; Treasurer, Leroy Webber, Rochester. The meeting then adjourned until 2 P.M At the afternoon session came the reading of papers.

Dr. Taylor's paper on "Acute Indigestion and Flatulent Colic" was a very excellent one, and was well received and discussed.

Dr. Kesler opened the discussion on the use of external applications in various diseases, which brought forth a considerable interchange of ideas, which was very interesting.

Dr. N. N. Loefler's subject, "Nasal Gleet," in which he reported a case of very complete impaction of the sinuses, with a thick cheesy pus.

Dr. P. I. Johnson presented a specimen of rupture of the extensor pedis tendon in a young colt, two days old. It was an excellent specimen and well prepared.

After a lively discussion on various other subjects relating to the profession, the meeting adjourned to meet in July.

W. E. Stocking,
Secretary.

### CONNECTICUT VETERINARY MEDICAL ASSOCIATION.

The annual meeting of the Association was held at Hartford on Tuesday, February 4, 1902.

President E. C. Ross, of New Haven, called the meeting to order at 3 o'clock, and the session continued until 6 o'clock, when a short recess was taken.

Roll-call was answered by eighteen veterinarians, as follows: Dr. Thomas Bland, P. T. Keeley, and J. E. Devereaux, of Waterbury; Dr. E. C. Ross, F. G. Atwood, H. Whitney, of New Haven; Dr. Andrew Hyde, of Norwich; Dr. G. H. Parkinson, of Middletown; Dr. L. B. Judson, of Winsted; Drs. G. F. McGuire, G. T. Crowley, and C. R. Witte, of New Britain; Dr. B. K. Dow, of Willimantic; Drs. R. P. Lyman, F. A. Ingram, P. F. Finnegan, of Hartford; Dr. T. A. Donaldson, of Manchester, and Dr. H. E. Bates, of South Norwalk.

Application for membership was received and favorably acted upon by the Association for Drs. E. H. Lenhert, of Storrs Agricultural College, and Thomas Laden, of New Haven.

Dr. L. Y. Ketcham, formerly of South Woodstock, now of California, asked for a demit, which was granted. Upon proper motion to the effect, the President instructed the Secretary to draft resolutions on the death of Dr. R. S. Huidekoper, of Philadelphia, and also on the death of Dr. W. H. Prophett, of Suffield, and to cause a copy of these resolutions to be sent to the Journal of Comparative Medicine and Veterinary Archives, to the *Veterinary Review*, and also to be spread upon the minutes of this meeting.

The resolutions are as follows:

Whereas, The Connecticut Veterinary Medical Association has learned with profound sorrow of the death of Dr. Rush S. Huidekoper, of Philadelphia, an honored member of the veterinary profession, a man who labored earnestly to improve the veterinary service in the United States army, and devoted his life to the elevation of his chosen field of labors;

*Resolved*, That by his death the field of journalism has lost a valued helper; and be it further

*Resolved*, That this Association mourns his loss, and causes a copy of these resolutions to be sent to the Journal of Comparative Medicine and Veterinary Archives, the *Veterinary Review*, and also be spread upon the records of this Association.

WHEREAS, The Connecticut Veterinary Medical Association has lost, in the death of Dr. William H. Prophett, of Suffield, one of its older members; be it

*Resolved*, That this Association hereby mourns his loss; and be it further

*Resolved*, That a copy of the resolutions be sent to the *Veterinary Journal* and *American Veterinary Review*, and also be spread upon the records of this Association.

Neither Dr. E. C. Ross, of New Haven, the President, nor Dr. R. P. Lyman, of Hartford, the retiring Secretary, could be induced to accept a renomination.

The election of officers resulted as follows:

President: Dr. Andrew Hyde, of Norwich.

Vice-Presidents: Dr. Thomas Bland, of Waterbury, and Dr. H. Whitney, of New Haven.

Secretary: Dr. B. K. Dow, of Willimantic.

Treasurer: Dr. E. C. Ross, of New Haven.

Board of Censors: Drs. H. E. Bates, South Norwalk; P. T. Keeley, Waterbury; F. A. Ingram, Hartford; G. F. McGuire, of New Britain; and Dr. R. D. Martin, of Bridgeport.

After reassembling, the members listened to a carefully prepared paper by Dr. A. Hyde on "Milk Inspection." Other papers by Dr. J. E. Gardner, of Hartford; Dr. F. G. Atwood, of New Haven; and Dr. Crowley, of New Britain, were postponed owing to lateness of the hour.

Prior to adjournment it was voted to hold the semi-annual meeting at Hartford on the first Tuesday in August.

ANDREW HYDE,
President.

B. K. DOW,
Secretary.

### THE VETERINARY ASSOCIATION OF MANITOBA.

THIS Association held its twelfth annual meeting in the city of Winnipeg, February 19th. The President, Mr. W. A. Dunbar, occupied the chair, and the following members were present: W. H. Smith, of Carman; W. R. Taylor, Portage la Prairie; W. J. Hinman, Winnipeg; H. F. Whaley, Glenboro; G. W. Harrison, Cypress; J. McGillivray, Manitou; W. S. Henderson, Carberry; W. Swenerton, Carberry; J. J. Irwine, Stonewall; J. G. Cruikshank, Deloraine; J. Golley, Treherne; C. D. McGilvray, Binscarth; J. Welch, Roland; S. A. Cox, Brandon; J. A. Stevenson, Carman; W. E. Martin, Winnipeg; A. M. Livingstone, Melita; M. Whimster, Hamiota; W. A. Hilliard, Minnedosa; R. D. Scurfield, Crystal City; W. A. Dunbar, F. Torrance, H. D. Smith, C. Little, Winnipeg; D. D. Reid, Hartney; and as visitors, Drs. Simpson, of Yorkton, and Sankey, of Waskada.

The President opened the meeting with a few words of welcome to the members and visitors, and then read an interesting and instructive address upon the progress of veterinary science.

A letter from the Secretary of the Winnipeg Humane Society was read, asking the views of the Association upon the overhead check. After a full discussion of the subject, it was moved by W. A. Dunbar, seconded by Mr. J. A. Stevenson, that this Association, while deploring the fact that some cruelty is inflicted upon horses by excessively high checking with the overhead check, and anxious to do all in its power to mitigate the evil, also admits that the said check can be used without inflicting pain, and in some cases, such as kickers and hard-pullers, is absolutely necessary for their control and the safety of their drivers, and the Secretary is hereby instructed to reply to the Humane Society in the terms of this resolution; carried.

The Resident Secretary of the A. V. M. A. called the attention of the members to the fact that the next meeting of that Association would be held in Minneapolis during the first week in September. Reduced fares would be obtainable from the railways, and all who went could depend upon having a profitable and enjoyable visit. He hoped a large number would take the opportunity.

The Secretary-Treasurer read his annual report, showing a membership of seventy-five and a surplus in the treasury of some four hundred dollars.

The examiners reported that during the year four candidates presented themselves for examination, of which two were successful—Mr. C. D. McGilvray, of Binscarth, and Mr. R. D. Scurfield, of Crystal City, both graduates of the McKillip Veterinary College, of Chicago.

The election of officers for the ensuing year resulted as follows:

President: S. A. Coxe, of Brandon.

Vice-President: A. M. Livingstone, Melita.

Secretary-Treasurer and Registrar: F. Torrance, Winnipeg.

Examiners: W. A. Dunbar, W. R. Taylor, and F. Torrance.

Other members of Council: W. S. Henderson and W. Swenerton, of Carberry.

Auditors: C. Little, W. E. Martin.

The Association passed unanimously the following resolution congratulating Dr. Rutherford upon his recent appointment:

"*Resolved*, That this Association rejoices in the elevation of one of its members to the most important post in the Dominion open to the veterinary profession, that of Chief Veterinary Inspector to the Department of Agriculture, and wishes to place on record its appreciation of Dr. Rutherford's work as a founder of this Association and as one of its most active members, and hereby tenders him its heartiest congratulations, and wishes him every success in his new sphere."

The Association also, by unanimous vote, elected Dr. Rutherford an Honorary Associate.

A resolution was passed to memorialize the Dominion Government to appropriate a sum of money for the investigation of the disease of horses commonly known as "swamp fever," which is continuing to cause great losses in parts of Manitoba and the Northwest Territories.

Dr. W. A. Hilliard read a paper upon an interesting surgical case occurring in his practice. An animated discussion followed, in which the subject of mallein test was also brought up and some interesting experiences related.

In the hope of inducing the presentation of a larger number of papers at the next meeting the following resolution was passed:

Moved by W. A. Dunbar, seconded by J. A. Stevenson, that three prizes, to consist of books or instruments, be offered for competition for the best essays or reports of cases presented at the annual meeting, competition limited to members who have never read a paper before the Association. The meeting to decide on the merits of the papers; carried.

After the usual vote of thanks to the retiring President, the essayist, and the City Council, the meeting adjourned.

The semi-annual meeting will be held in Brandon in July.

F. TORRANCE,
Secretary.

### PHILIPPINE VETERINARY MEDICAL ASSOCIATION.

THE veterinarians of the Philippine Islands, to the number of fourteen, assembled at Manila recently and organized a veterinary association under the above name. Most of these members of the profession are from the States, and are located in and about Manila among our American army posts.

The following officers were chosen: President, Dr. W. R. L. Best; Vice-President, Dr. T. H. Tucker; Secretary, Dr. W. W. Richards; and Treasurer, Dr. H. H. Mercke. A constitution and by-laws were adopted and meetings arranged for once a month. A Board of Censors were selected as follows: Drs. Sorenson, Randall, and McKenzie. A Committee on Diseases, consisting of Drs. Hill, Andre, Smith, and Slee. A Committee on Finance, consisting of Drs. Carroll, Randall, and Sorenson. This Association should be of much interest to English-speaking and reading veterinarians as to the diseases that may be prevalent there and possibly transferred to the United States through the intimate commercial relations now being established.

